# The Impact of Parental Styles on the Development of Psychological Complaints

**DOI:** 10.5964/ejop.v11i1.836

**Published:** 2015-02-27

**Authors:** Daniela Rocha Lopes, Kees van Putten, Peter Paul Moormann

**Affiliations:** a Psychologist, Sao Paulo, Brazil; b Department of Methodology, Faculty of Social Sciences, Leiden University, Leiden, The Netherlands; c Department of Clinical, Health and Neuropsychology, Faculty of Social Sciences, Leiden University, Leiden, The Netherlands; Aalborg University, Aalborg, Denmark

**Keywords:** Rogers, parental styles, psychological complaints, neuroticism, conditional regard

## Abstract

The main aim of the present study was to test Rogers’ theory, stating that parental styles characterized by unconditional positive regard (UPR) promote healthier adults than parental styles characterized by conditional regard (CR). For both caregivers CR was found to be associated with significantly higher scores on psychological complaints than UPR (on nearly all SCL-90 scales and the SCL-total score), even when controlling for gender. Although lack of emotional warmth by the father and harsh discipline by the mother were significant predictors of SCL-90-Total (indicating state neuroticism) it should be noted that both variables only explained a small amount of the total variance. Empirical evidence was found for Rogers’ theory. Others factors than merely emotional warmth and discipline play a role in the etiology of state neuroticism. For future research it is therefore recommended to include other factors, such as daily worries, temperament, and alexithymia

## Introduction

The role of early experiences in the development of future psychological problems has been the focus of many personality theories, as demonstrated in Rogers’s theory (1951), which mainly considered that a healthy development of personality has to do with favorable conditions which occur especially in parent-child interactions during the formative years. In his theory, the considered optimal relationship is Unconditional Positive Regard (UPR), which is characterized by the child being consistently prized regardless of one’s behavior, and moreover by constantly receiving affection and acceptance. Rogers (1951) also described the considered less optimal parental style, Conditional Regard (CR), which on the other hand, is characterized by a tendency of the child to perceive that one is only valued when one’s behavior is in accordance to the parents expectancy since parent-child interactions are permeated by inconsistencies both in discipline and in expressions of love. Roger’s concept of CR (1951) has features in common with later constructs, such as Parker’s concept of Affectionless Control (AC) rearing style (1990), characterized by harsh discipline in combination with the lack of a loving attitude. Parker (1990) stated that AC parental style seemed to be a risk factor for neurosis or simply for seeking for help.

The pathway from UPR to psychological health can be understood through the presence of parents’ consistency in the promotion of emotional warmth and discipline leading to the development of more secure individuals. Individuals whose parents demonstrated support, affection and responsiveness to their needs most of the time tend to be less sensitive to rejection. In the same way, they are also more inclined to see themselves as more competent and likeable (Wearden, Peters, Berry, Barrowclough, & Liversidge, 2008). Therefore, it is possible to assume that the presence of consistency in the way parents rear their children directly affect in a positive way the way they see themselves. In that sense, research in this field suggests that individuals reared in UPR parental styles tend to present a higher self-concept. Due to this condition, those individuals could experience less vulnerability either from negative internal or external cues. On the other hand, individuals with a higher sense of vulnerability tend to experience more psychological distress when exposed to negative internal or external cues.

AC, on the other hand, is more related to the presence of parental inconsistencies in rearing styles. Inconsistencies were shown to be predictors of psychological disorders because they promote ambivalent feelings towards the self, unpredictability as aversive stimuli and feelings of being subjected to injustice (Dwairy, 2010).

As it has been mentioned above, AC parental style can be compared to the notion of Conditional Regard, which was demonstrated to cause an unstable sense of self a fragile self-esteem and a sense the one’s self worth is vulnerable (Kernis et al., 1998). The reason why CR or AC parental style might be associated with lack of psychological health can be attributed to parents’ use of love withdrawal and guilt induction (Kernis, Brown, & Brody, 2000) as important factors generating unstable self-esteem.

### Parental Styles and Psychological Complaints

Several studies demonstrated the relation between negative parental styles and the occurrence of psychological complaints later in life.

Those studies demonstrated that individuals who were exposed to parental styles involving low levels of emotional warmth and high levels of inconsistent and punitive discipline demonstrated a higher probability of developing psychological complaints in the future such as agoraphobia (Manicavasagar, Silove, Wagner, & Hadzi-Pavlovic, 1999), depression and anxiety (McGinn, Cukor, & Sanderson, 2005), low self-esteem (Schlette et al., 1998), somatic complaints (Lackner, Gudleski, & Blanchard, 2004), hostility (Muris, Meesters, Morren, & Moorman, 2004) and sleepiness (Brand, Hatzinger, Beck, & Holsboer-Trachsler, 2009).

### Maternal and Paternal Styles and Psychological Complaints

Most studies in this field focused more on the maternal figure, by presenting data supported either exclusively by the analyses of the maternal style or by sample selection based on having equal maternal and paternal styles (Simons & Conger, 2007).

Previous studies which have taken in consideration both paternal and maternal rearing styles reported different results concerning a higher influence of the father, of the mother, or even of both parents on the development of psychological symptoms (Gerlsma, Emmelkamp, & Arrindell, 1990; Xia & Qian, 2001).

The present study makes a novel contribution to the existing literature by analyzing parental styles through the presence or lack of two factors combined: emotional warmth and discipline. Moreover, the research focuses not only on specific psychological complaints but mainly in their general consequences, known as the concept of neuroticism, in particular its state component. Finally, the present study aims to contribute to relevant findings regarding the influence of the father’s role in shaping personalities of children from child towards adulthood (Khaleque & Rohner, 2012).

### Hypotheses

The present study will examine whether early experiences of CR (Rogers, 1959) rearing style, which is characterized by high scores on discipline (inconsistent punishment, harsh discipline) in combination with low scores on emotional warmth (lack of a loving attitude), lead to more psychological complaints in adulthood than early experiences of a parental style characterized by UPR (Rogers, 1959), which is characterized by low scores on discipline (consistent punishment, positively oriented discipline) in combination with high scores on emotional warmth (loving attitude). This will be done for paternal and maternal parenting styles separately. Differences between CON (high on discipline and high on emotional warmth) and IND (low on discipline and low on emotional warmth) parental styles, will be explored as well. Furthermore the impact of the respondent’s gender will be investigated. Finally, it will be tested whether the dimensions of discipline and emotional warmth in parental styles predict psychological complaints.

Considering that inconsistencies present in CR parental style were revealed to be important factors generating unstable self-esteem (Kernis et al., 2000) it is expected that subjects exposed to CR parental style present more psychological complaints and, therefore a higher level of neuroticism than those exposed to UPR parental styles.

## Material and Methods

### Participants

A total of 263 undergraduates in Psychology of Leiden University (52 males; *M*_age_ = 23.32, and 211 females; *M*_age_ = 22.73) participated in our research. When tested, none of the students was familiar with the instruments used or with the aim of the study.

### Procedure

The LPCIQ and the SCL-90-Dutch Version were administered on the Mental Information Processing and Neuropsychological Diagnostic System (MINDS'96 Test Manager, (Brand, 1996; Brand & Houx, 1992). All questionnaires were part of a much larger test battery, in which personality questionnaires and neuropsychological tests were included.

### Instruments

Psychological complaints will be measured through the Dutch adaptation of the SCL-90 (Arrindell & Ettema, 1986), whereas parental styles will be measured for both father and mother separately through the LPCIQ-Revised (Moormann, Bermond, Albach, & Van Dorp, 1997; Moormann, de Cocq van Delwijnen, van Wessel, & Bauer, 1989; Moormann & Van Dijk, 1992).

*LPCIQ-R* consists of 21 items and has two dimensions of which the reliability coefficients are: (1) emotional warmth (α_father-child_ = .89; α_mother-child_ = .88) and (2) discipline (α_father-child_ = .85; α_mother-child_ = .85). Parental styles were constructed by combining extreme scores (> 70^th^ percentile and < 30^th^ percentile) on the Emotional Warmth (EW) and Discipline (D) dimensions, resulting in four parenting styles: CR (low on EW, high on D), UPR (high on EW, low on D), CON (high on EW, high on D) and IND (low on EW, low on D).

*SCL-90–Dutch adaptation* consists of nine scales measuring recently experienced somatic and psychological complaints. The main dimension of the SCL-90 is its total score (state measure of neuroticism), based on the sum total of the following scales: agoraphobia, anxiety, depression, somatic complaints, insufficiency of thinking and acting, interpersonal sensitivity, hostility and sleep problems. Reliability and validity are good (Evers, Van Vliet-Mulder, & Ter Laak, 2000).

## Results

As parental styles were constructed by combining extreme scores (based on percentile scores) on the Emotional Warmth and Discipline dimensions of the LPCIQ-R only a selection of the respondents were part of the ANOVAs. The cut off scores for the father were: > 41 on EW and < 16 on D for UPR; < 35 on EW and > 18 on D for CR; > 41 on EW and > 18 on D for CON; < 35 on EW and < 16 on D for IND. The cut off scores for the mother were: > 44 on EW and < 20 on D for UPR; < 39 on EW and > 22 on D for CR; > 44 on EW and > 22 on D for CON; < 39 on EW and < 20 on D for IND.

Our main hypothesis was that CR (both paternal and maternal) leads to significantly higher scores on psychological complaints than UPR. Descriptives of the SCL-90 variables for the four paternal and maternal parental styles are reported in Tables 1a and 1b.

**Table 1a:** Descriptives for the Parental Styles of Father Concerning Psychological Complaints (Based on the Scores From all SCL-90 Scales)

Scale	UPR			CR			CON			IND		
	*M*	*SD*	*n*	*M*	*SD*	*n*	*M*	*SD*	*n*	*M*	*SD*	*n*
Agoraphobia	7.33	0.93	58	8.41	3.11	51	7.05	0.23	19	7.40	0.74	15
Anxiety	12.52	3.96	58	14.47	4.42	51	12.89	4.00	19	12.73	2.15	15
Depression	20.69	6.23	58	26.31	10.84	51	23.32	10.68	19	24.53	9.08	15
Somatic	15.66	3.78	58	17.24	5.19	51	15.16	4.90	19	17.27	3.56	15
Insufficiency	12.17	3.17	58	15.94	6.68	51	13.11	3.71	19	13.13	2.95	15
Sensitivity	21.84	3.65	58	28.73	12.14	51	22.95	5.05	19	22.67	4.47	15
Hostility	6.98	1.16	58	7.84	2.74	51	7.84	3.91	19	8.00	3.07	15
Sleep	3.78	1.30	58	4.69	2.34	51	4.89	2.64	19	3.87	0.99	15
Neuroticism	111.41	17.70	58	136.49	43.32	51	118.37	33.45	19	120.20	20.63	15

### Results of Psychological Complaints for the Parental Style Father

The results of the one-way ANOVAs with all SCL-90 scales as dependent variables and parental styles of the father as independent variable were significant for agoraphobia, *F*(3, 139) = 3.69, *p* = .013, depression, *F*(3, 139) = 3.61, *p* = .015, insufficiency, *F*(3, 139) = 5.88, *p* = .001, sensitivity, *F*(3, 139) = 7.40, *p* < .001, sleep problems, *F*(3, 139) = 2.99, *p* = .033, and the Neuroticism scale, *F*(3, 139) = 5.89, *p* = .001. The strength of the relationship between parental styles of the father and agoraphobia, depression, insufficiency, sensitivity, sleep problems, and Neuroticism as assessed by *Partial Eta Squared*, varied from medium (.06) to large (.14) in the cases above, with the level factor accounting from 6% to 14% of the variance of the dependent variable. The *Levene’s Test of Equality of Error variances* was only non-significant for somatic complaints. Therefore contrast tests not assuming equal variances were used for agoraphobia, depression, insufficiency, sensitivity, sleep problems, and Neuroticism. The contrast coefficients for the main hypothesis were -1, 1, 0, 0 for UPR, CR, CON and IND, respectively. All t-tests were one-tailed.

The results of the contrasts between UPR and CR were significant for agoraphobia, *t*(57.76) = 2.40, *p* = .01, depression, *t*(77.52) = 3.26, *p* = .001, insufficiency, *t*(69.42) = 3.68, *p* < .001, sensitivity, *t*(57.95) = 3.90, *p* < .001, sleep problems, *t*(75.93) = 3.70, *p* = .008, and Neuroticism, *t*(64.55) = 3.86, *p* < .001.

With the exception of anxiety, somatic complaints and hostility all other scales confirmed the main hypothesis for the father. On the whole respondents with fathers practicing UPR displayed significantly lower psychological complaints than fathers practicing CR.

**Table 1b:** Descriptives for the Parental Styles of Mother Concerning Psychological Complaints (Based on the Scores From all SCL-90 Scales)

Scale	UPR			CR			CON			IND		
	*M*	*SD*	*n*	*M*	*SD*	*n*	*M*	*SD*	*n*	*M*	*SD*	*n*
Agoraphobia	7.33	0.99	33	8.63	3.43	43	7.38	0.88	24	7.44	1.04	25
Anxiety	12.82	3.21	33	15.23	5.94	43	12.29	1.71	24	12.68	2.78	25
Depression	21.48	5.30	33	28.98	13.86	43	21.63	6.06	24	22.44	6.21	25
Somatic	15.76	3.83	33	17.63	6.17	43	15.79	3.61	24	15.56	2.95	25
Insufficiency	12.73	3.38	33	15.77	6.40	43	13.71	4.25	24	12.88	3.63	25
Sensitivity	22.48	3.93	33	27.33	11.66	43	23.25	6.00	24	23.84	6.48	25
Hostility	6.85	1.52	33	8.07	3.01	43	6.75	1.23	24	6.88	1.24	25
Sleep	3.85	1.68	33	4.51	2.21	43	3.88	1.33	24	4.04	1.40	25
Neuroticism	114.03	17.13	33	139.23	46.49	43	115.29	20.68	24	116.76	20.66	25

### Results of Psychological Complaints for the Parental Style Mother

The results of the one-way ANOVAs with all SCL-90 scales as dependent variables and parental styles of the mother as independent variable were significant for agoraphobia, *F*(3, 121) = 3.13, *p* = .028, anxiety, *F*(3, 121) = 3.84, *p* = .011, depression, *F*(3, 121) = 5.48, *p* = .001, insufficiency, *F*(3, 121) = 3.17, *p* = .027, hostility, *F*(3, 121) = 3.35, *p* = .021, and the Neuroticism scale, *F*(3, 121) = 5.49, *p* = .001. The strength of the relationship between parental styles of the mother and agoraphobia, anxiety, depression, insufficiency, hostility, and Neuroticism as assessed by *Partial Eta Squared*, was medium in all cases, with the level factor accounting from 6% to 12% of the variance of the dependent variable. The *Levene’s Test of Equality of Error variances* was only non-significant for sleep problems. Therefore contrast tests not assuming equal variances were used for agoraphobia, anxiety, depression, insufficiency, hostility, and Neuroticism. The contrast coefficients for the main hypothesis were -1, 1, 0, 0 for UPR, CR, CON and IND, respectively. All *t*-tests were one-tailed.

The results of the contrasts between UPR and CR were significant for agoraphobia, *t*(50.82) = 2.35, *p* = .01, anxiety, *t*(67.25) = 2.27, *p* = .01, depression, *t*(56.84) = 3.25, *p* = .001, insufficiency, *t*(66.50) = 2.67, *p* = .005, hostility, *t*(65.17) = 2.30, *p* = .012, and Neuroticism *t*(55.88) = 3.28, *p* = .001.

With the exception of somatic complaints, sleep problems and sensitivity all other scales confirmed the main hypothesis for the mother. On the whole respondents with mothers practicing UPR displayed significantly lower psychological complaints than mothers practicing CR.

### Beyond the Main Hypotheses

In addition to CR and UPR parental styles, possible differences considering Indifferent and Confusing parental styles were also investigated using a one-way ANOVA with post-hoc tests. The *Levene tests of homogeneity of variances* for the father regarding somatization *p* = .24 showed that the Bonferroni post-hoc test, assuming equal variances could be used. For agoraphobia (*p* = .00), anxiety (*p* = .03), depression (*p* = .00), insufficiency (*p* = .00), sensitivity (*p* = .00), hostility (*p* = .00), sleep problems (*p* = .00), and Neuroticism (*p* = .00) Dunnet's C was used, which does not assume equal variances. CR resulted in significant higher scores (*p* = .05, two-tailed) than CON for sensitivity and agoraphobia. Moreover CR resulted in significant higher scores (*p* = .05, two-tailed) than IND for sensitivity (see Table 1a).

The *Levene tests of homogeneity of variances* for the mother regarding sleep problems (*p* = .26) showed that the Bonferroni post-hoc test, assuming equal variances could be used. For agoraphobia (*p* = .00), anxiety (*p* = .03), depression (*p* = .00), somatic complaints (*p* = .01), insufficiency (*p* = .04), sensitivity (*p* = .01), hostility (*p* = .00), and Neuroticism (*p* = .00) Dunnet's C was used, which does not assume equal variances. CR resulted in significant higher scores (*p* = .05, two-tailed) than CON for Neuroticism, anxiety and depression. Moreover CR resulted in significant higher scores (*p* = .05, two-tailed) than IND for Neuroticism (see Table 1b).

The overall picture for both paternal and maternal parenting styles shows the negative effect of CR.

### Gender Differences

Gender differences were found, except for sensitivity, hostility and sleep problems for all SCL-90 scales, including Neuroticism. Descriptives are given in Table 2. The *Levene’s test* was significant for all SCL-90 variables, except for sensitivity, hostility and sleep problems. It should be noted that the number of respondents is based on the whole file, e.g. all respondents that filled out the SCL-90.

**Table 2:** Overview of the Scores on SCL-90 Scales Sorted by Gender

Gender	*n*	*M*	*SD*	*t*	*p* (2-tailed)
Neuroticism					
Female	812	123.28	30.07	*t*(356.53) = 3.29	.001
Male	192	116.74	23.40		
Agoraphobia					
Female	812	7.73	1.78	*t*(495.04) = 2.54	.011
Male	192	7.48	1.03		
Anxiety					
Female	812	13.34	3.89	*t*(335.24) = 2.70	.007
Male	192	12.61	3.23		
Depression					
Female	812	23.72	8.47	*t*(359.05) = 4.17	.000
Male	192	21.39	6.55		
Somatic					
Female	812	16.65	4.51	*t*(335.09) = 4.88	.000
Male	192	15.12	3.74		
Insufficiency					
Female	812	13.90	4.50	*t*(360.00) = 2.04	.042
Male	192	13.29	3.47		
Sensitivity					
Female	812	24.50	7.26	*t*(320.45) = 1.06	.290
Male	192	23.94	6.34		
Hostility					
Female	812	7.53	2.22	*t*(307.56) = 1.39	.166
Male	192	7.30	2.04		
Sleep					
Female	812	4.44	2.05	*t*(303.15) = 0.26	.799
Male	192	4.40	1.92		

The parenting styles imply that only extreme scores are used. Therefore the number of respondents is much lower for the analyses where differences between parental styles were investigated than the number of respondents involved in testing for sex differences. However the results from Table 2 suggest that is advisable to control for gender differences, because on most SCL-90 scales female respondents scored significantly higher than male respondents. Considering the large number of ANCOVAs (eighteen in total) that had to be performed to control for gender first a data reduction technique was applied, e.g. a Principal Component Analysis (PCA) was performed on the SCL-90 subscales, to see how they were interrelated. The main issue was how many components could be distinguished among the SCL-90 complaints. The *PCA* (see Table 3) with Varimax rotation, conducted on the 8 SCL-90 subscales, resulted in a one-factor solution, accounting for 56.0% of the total variance. Component 1 with an Eigenvalue of 4.5 accounted for 56.0% of the variance.

**Table 3:** PCA on the 8 SCL-90 Subscales

Scale	Component 1: Neuroticism
Agoraphobia	0.64
Anxiety	0.83
Depression	0.87
Somatic	0.70
Insufficiency	0.84
Sensitivity	0.82
Hostility	0.69
Sleep	0.55

The results from the PCA clearly indicated that there was only one common factor underlying the various SCL-90 complaints. This factor is called Neuroticism (acknowledged by the test constructors and composed of the sum total of all the 8 subscales; see Arrindell & Ettema, 1986).

When considering the result from the PCA (the subscales are highly interrelated) only 2 ANCOVAs were performed instead of 18, where the sum total score of the subscales (Neuroticism) acted as dependent variable, the paternal and maternal parenting styles (CR, UPR, CON and IND) as independent variable and gender as covariate.

### Gender Differences in Psychological Complaints by Parental Style Father

A one-way analysis of covariance (ANCOVA) was conducted in order to eliminate the effect of gender as a potential confounding. A preliminary analysis evaluating the homogeneity-of-slopes assumption for the parental style by the father indicated that the relationship between the covariate (gender) and the dependent variable (Neuroticism score) did not significantly differ as a function of the independent variable (parental style by the father), *F*(3, 135) = .76, *MSE* = 732.97, *p* = .52, partial η^2^ = .02. The ANCOVA was significant, *F*(3, 138) = 6.39, *MSE* = 964.84, *p* < .01. The strength of the relationship between the parental style by the father and the Neuroticism score was medium, as assessed by a partial η^2^, with the parental style by the father accounting for 12% of the variance of the dependent variable, holding constant the gender factor. Therefore differences on the Neuroticism scores among groups did not vary as a function of gender.

Gender was significant for Neuroticism (*p* = .03) and the parental style by the father was also significant (*p* < .01). These data show that although gender was significant, the impact of the overall parental styles is much stronger than a possible influence by gender on Neuroticism. Therefore the parental style by the father was shown to be determinant for the scores on Neuroticism and gender could be eliminated as a possible confounding since it was shown that being either male or female as a respondent did not play a crucial role on Neuroticism scores.

### Gender Differences in Psychological Complaints by Parental Style Mother

Again a one-way analysis of covariance (ANCOVA) was conducted in order to eliminate the effect of gender as a potential confounding. A preliminary analysis evaluating the homogeneity-of-slopes assumption for the parental style by the mother indicated that the relationship between the covariate (gender) and the dependent variable (Neuroticism score) did not significantly differ as a function of the independent variable (parental style by the mother), *F*(3, 117) = .15, *MSE* = 153.62, *p* = .93, *η^2^* = .00. The ANCOVA was significant, *F*(3, 120) = 4.20, *MSE* = 982.18, *p* = .01. The strength of the parental style by the mother and the Neuroticism score was medium, as assessed by a partial η^2^, with the parental style by the mother accounting for 9% of the variance of the dependent variable, holding constant the gender factor. Therefore differences on the Neuroticism scores among groups did not vary as a function of gender.

Gender was not significant for Neuroticism (*p* = .12), but the parental style by the mother was significant (*p* = .01). Once again these data show that the impact of the overall parental styles is much stronger than a possible influence by gender on Neuroticism. Therefore the parental style by the mother was shown to be determinant for the scores on Neuroticism and gender could be eliminated as a possible confounding since it was shown that being either male or female did not play a crucial role on Neuroticism scores.

In conclusion, the analyses performed when controlling for gender, demonstrated that the overall effect of both paternal and maternal parenting styles is much stronger than gender.

### Results of Parental Style Dimensions (Emotional Warmth and Discipline) on Psychological Complaints

A stepwise multiple regression analysis was carried out, where the four parental style dimensions (Emotional Warmth by the Father, Emotional Warmth by the Mother, Discipline by the Father and Discipline by the Mother) were entered as predictor variables and Neuroticism (SCL-90Total Score) as dependent variable. The results are given in Table 4.

**Table 4:** Stepwise Multiple Regression Analysis Predicting the Neuroticism Score (SCL-90 Total Score) From Both Paternal and Maternal Emotional Warmth and Discipline Components of the LOK-R

Scale	beta	r	R	Adj. R square
Emotional Warmth Father	-0.23***	-0.23	-0.23	0.05
Discipline Mother	0.12*	0.13	0.13	0.06

*p < .05. ***p < .001.

The outcome of the stepwise multiple regression analysis indicated that the state Neuroticism scale (SCL-90 Total Score) was predicted from lack of Emotional Warmth by the Father and harsh Discipline by the Mother. The two predictor variables together explained only 6% of the total variance. Lack of Emotional Warmth by the father was the strongest predictor. The dimensions of Emotional Warmth by the Mother and Discipline by the Father were not significant.

## Discussion

The main outcome supports Roger’s notion (1951) that CR compared with UPR would lead to significantly more psychological distress later in life. With the exception of a few scales respondents with CR parents reported significantly higher scores on nearly all psychological complaints, including the sum total of the SCL-90 (state neuroticism) than respondents with UPR parents. This result held for both paternal and maternal parental styles. The devastating effect of CR remained significant even when other paternal and maternal parental styles (e.g. Confusing and Indifferent) were involved in the analyses. Those findings are also in accordance with previous studies showing that negative parenting styles led to more psychological complaints than positive parenting styles (Brand et al., 2009; Maynard & Harding, 2010; McGinn et al., 2005).

Although there were some differences in the way male and female respondents reported symptoms of distress, parental styles demonstrated to have more importance than respondent’s gender in what refers to the impact they have on psychological complaints.

A remarkable finding was the importance that must be given to the role of the father. Both the lack of emotional warmth by the father and the harsh discipline by the mother were found to be the main predictors of state neuroticism. As the lack of emotional warmth by the father explained a greater amount of the total variance than harsh discipline by the mother our data seem to suggest that the role of the father should not be overlooked as a primary caregiver in the development of psychological complaints in the offspring. A cold father was the one exerting the strongest impact on psychological complaints.

It has been shown that although the amount of time spent with the children was not predicted by the sex of the parent, mothers still reported to take significantly more responsibility for child-related tasks and for disciplining, regardless of mothers also having a professional career (Renk et al., 2003). It has also been found that fathers experienced more satisfaction than mothers with their parenting responsibilities probably because fathers were usually involved in more leisure activities than in assisting in child-related tasks (Renk et al., 2003). Through these findings it is possible to assume that parents having a better division regarding childcare would not only allow women to reduce their stress and increase their satisfaction regarding parenting responsibilities, but also possibly provoke an increase in fathers’ emotional involvement with the offspring, promoting therefore a healthier psychological development of those individuals.

Our findings corroborate with Khaleque and Rohner (2012) research since father’s affection seem to be critical to a person’s development, contributing not only with a child’s development but being also responsible for shaping personalities throughout adulthood. The implication of this lies is the reassignment of a more egalitarian division of the importance of both caregivers, removing the emphasis that is often attributed to the maternal role. Therefore, this finding supports the importance of men becoming more involved in nurturing child care.

### Clinical Implications

Professionals designated to assist families and parents in educational and clinical settings should pay special attention to the quality of fathers’ active participation and emotional involvement in childcare tasks. Furthermore, regarding the maternal role, the focus should be in assuring that mothers do not exert too harsh discipline in the upbringing of the children.

### Limitations

Some limitations should be considered when interpreting our findings. First, the vulnerability of our research for retrospective contamination (respondents reporting psychological complaints are inclined to blame their parents for their deplorable psychological state). This could have affected the outcomes of our study as we are in fact dealing with *perceived* parental styles.

Second, since lack of Emotional Warmth by the father and harsh Discipline by the mother only accounted for 6% of the total variance, other factors than only parenting styles should be considered when explaining the etiology of psychological complaints. Demographic variables such as the socioeconomic status of the respondent may affect the experience of psychological complaints. The same holds for whether parents were married or divorced; whether there was a significant difference in the amount of contact with each parent; and whether parents were biological or adoptive. The respondents personality is another factor of importance. It may be argued that some individuals are more apt to report psychological complaints than others, irrespective of parental styles. Dumont (2010) for instance refers to some studies (Beer, Arnold, & Loehlin, 1998; Eysenck, 1990; Goldsmith, Buss, & Lemery, 1997) which concluded that patterns of child-rearing exert little impact in shaping the temperament of a child. Although the SCL-90 total score is not a direct measure of temperament it cannot be denied that its subscales anxiety and hostility do relate to temperament. But even then there is another hurdle to overcome: the SCL-90 total score is a state neuroticism score, and it could be questioned to what extent state measures are generalizable to trait measures, since the SCL-90 essentially measures breaks in normal function rather than personality or traits (Schmitz, Kruse, Heckrath, Alberti, & Tress, 1999). However, state and trait measures are not fully independent of each other (see for instance Mischel, 1968 on the person-situation controversy). Individuals with high neuroticism scores tend to display stronger levels of psychological distress in stress provoking situations than individuals with low neuroticism scores, while everybody, regardless of the trait level of neuroticism, will react with more psychological distress in stressful situations. The foregoing seems to justify why temperament should be considered as a predictor of psychological distress. Citing some studies (Riemann, Angleitner, & Strelau 1997; Tellegen et al., 1988), Dumont (2010) considers that it is possible to find that approximately 70 percent of the variance could be accounted to genetic determinants whereas 20 to 35 percent of it are due to environment. In the present research even less, e.g. only 6% of the total variance was explained by the environment. As the SCL-90 deals with recently experienced somatic and psychological complaints it seems likely that all kinds of daily worries, not investigated in our research, might explain a substantial part of the remaining total variance. The same holds for disorders of affect regulation, because alexithymia types with an impaired cognitive component (reduced ability to verbalize, identify and analyze emotions) create a condition for reporting psychosomatic complaints and appear to hamper psychological health (Moormann et al., 2008). Therefore, future research should be aimed at the analysis and control of these factors contributing to psychological complaints.

However it should be noted that the focus of our research was not on all possible factors that might lead to psychological distress, but instead on testing Rogers’s theory stating that UPR promotes healthier individuals later in life than CR.
